# Treatment-refractory cutaneous Rosai–Dorfman disease responsive to oral methotrexate and topical trametinib

**DOI:** 10.1016/j.jdcr.2023.06.038

**Published:** 2023-07-07

**Authors:** Christopher J. Fay, Dorsa Moslehi, Christopher Iriarte, Timothy M. Dang, Cesar A. Virgen, Eleanor Russell-Goldman, Nicole R. LeBoeuf

**Affiliations:** a Department of Dermatology, Brigham and Women’s Hospital, Boston, Massachusetts; b Center for Cutaneous Oncology, Dana-Farber Cancer Institute, Boston, Massachusetts; c Harvard Medical School, Boston, Massachusetts; d Department of Pathology, Brigham and Women’s Hospital, Boston, Massachusetts

**Keywords:** cutaneous Rosai-Dorfman disease, histiocytic disorders, MEK inhibitor, methotrexate, non-Langerhans cell histiocytosis, Rosai–Dorfman disease, trametinib

## Introduction

Rosai–Dorfman disease (RDD) is a benign non-Langerhans cell histiocytosis characterized by accumulation of S100-positive, CD68-positive, and CD1a-negative histiocytes within lymph nodes.^1^ The disease classically presents with fever, night sweats, weight loss, and painless bilateral cervical lymphadenopathy. Over 40% of patients with RDD have extranodal involvement, most commonly including orbital tissue (11%), the nasal cavity (11%), skin (10%), bone (5% to 10%), and the central nervous system (5%).^2^ In 3% of RDD cases, patients have cutaneous disease in the absence of nodal disease, which is termed cutaneous RDD (CRDD).^3^ Some authors have argued CRDD is a distinct clinical entity.^4^ The median age at diagnosis is 44 years old with female predominance; patients of Asian or Caucasian descent are most commonly affected.^4^^,^^5^ Skin lesions of CRDD tend to present as asymptomatic red-brown papules, plaques, or nodules over the cheeks or periorbital area, often bilaterally.^4^^,^^5^ There are no consensus guidelines for treatment due to rarity of the disease and poor response to most interventions. A review of 56 CRDD cases demonstrated a cure rate at 28.6%.^5^ Surgical excision may be the most effective therapy with cure rates as high as 80%.^5^ Some patients, however, may be refractory to surgery, or further excision can be associated with significant morbidity in cosmetically sensitive areas. Here, we present a case of progressive, ulcerating CRDD responsive to the combination of methotrexate and a topical MEK inhibitor, added to intralesional triamcinolone.

## Case

**Fig 1:**
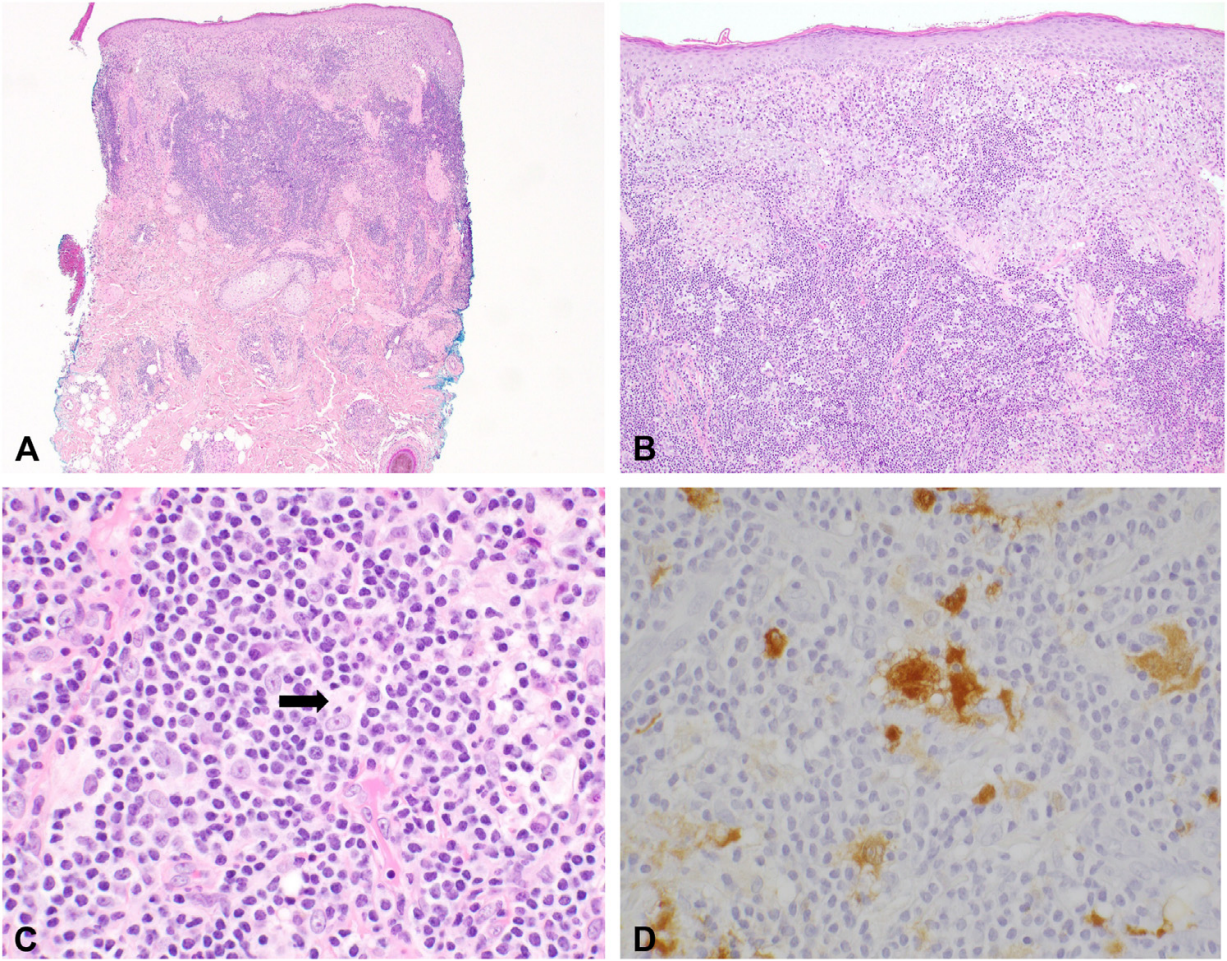
**A,** Superficial to mid-dermal mixed inflammatory infiltrate (H&E, 40× magnification). **B,** The inflammatory infiltrate consisted of numerous histiocytes. The epidermis was uninvolved (H&E, 100× magnification). **C,** Emperipolesis (*arrow*) and a background mixed inflammatory infiltrate composed of lymphocytes, plasma cells and neutrophils (H&E, 600× magnification). **D,** S100 immunostain highlights the lesional histiocytes and emperipolesis (60× magnification, S100 immunostain).

A 60-year-old male with no significant past medical history underwent excision of an asymptomatic cystic lesion over his right cheek that had been present for 3 months. Pathology revealed vacuolated histiocytes with prominent emperipolesis and an immunophenotype consistent with Rosai–Dorfman disease (Fig 1).

Examined margins were negative for residual disease. The patient was seen for interval follow-up during which exam was notable for a healing linear surgical scar without nodularity. No lymphadenopathy was appreciated clinically. Positron emission tomography-computed tomography (PET-CT) and brain magnetic resonance imaging (MRI) did not demonstrate extra-cutaneous disease or evidence of an associated malignancy. Laboratory workup revealed no abnormalities. Based on an unremarkable systemic evaluation, the patient was diagnosed with CRDD.

**Fig 2:**
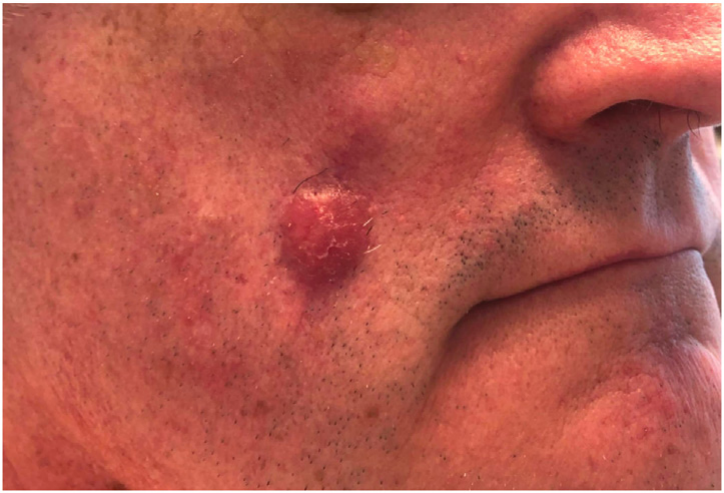
A *red-brown*, dome-shaped nodule over the *right* cheek at the site of prior surgical resection.

Four months later, the patient developed a well demarcated red-brown nodule at the site of the prior resection (Fig 2), as well as a new nodule over the left cheek.

Intralesional triamcinolone (ILTAC), at a concentration of 20 mg/mL, was administered to the right cheek nodule, and a punch biopsy of the left cheek nodule again confirmed CRDD. Growth slowed for 4 weeks, but growth persisted with no significant improvement after 4 months. The patient was then treated with topical imiquimod daily for 6 weeks followed by radiation therapy (20 Gray over 8 fractions).

A month after completion of radiation, exam was notable for bilaterally enlarging nodules. The patient continued imiquimod cream for 3 months with persistent growth, then 1 month of sirolimus cream twice daily.

**Fig 3:**
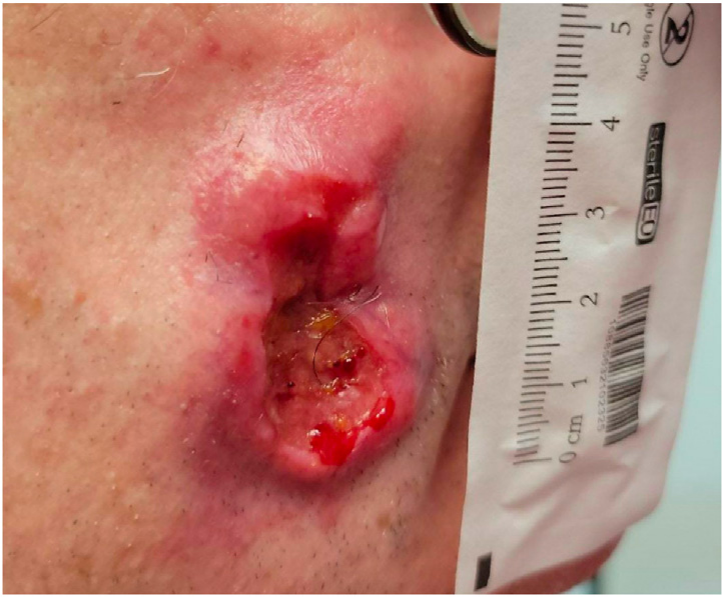
A large ulcerated thick plaque within the area of prior nodularity with surrounding reactive erythema and pinpoint bleeding.

The nodules continued to increase in size, eventually progressing to painful ulcerations (Fig 3).

Trametinib cream was compounded at a concentration of 1% with a lipoderm base (Chemistry Rx) and initiated twice daily with a plan for oral cobimetinib should lesions worsen, as histiocytic neoplasms are frequently driven by mutations in the mitogen-activated protein kinase (MAPK) pathway and the patient preferred trialing a targeted, topical alternative to oral MEK inhibition.^6^ With slow improvement of the superficial component of the disease over 2 months, the nodular deep component persisted and the patient was then started on oral methotrexate 25 mg weekly, while continuing trametinib cream and periodic intralesional triamcinolone (ILTAC). Growth halted and bleeding and pain both resolved.

**Fig 4:**
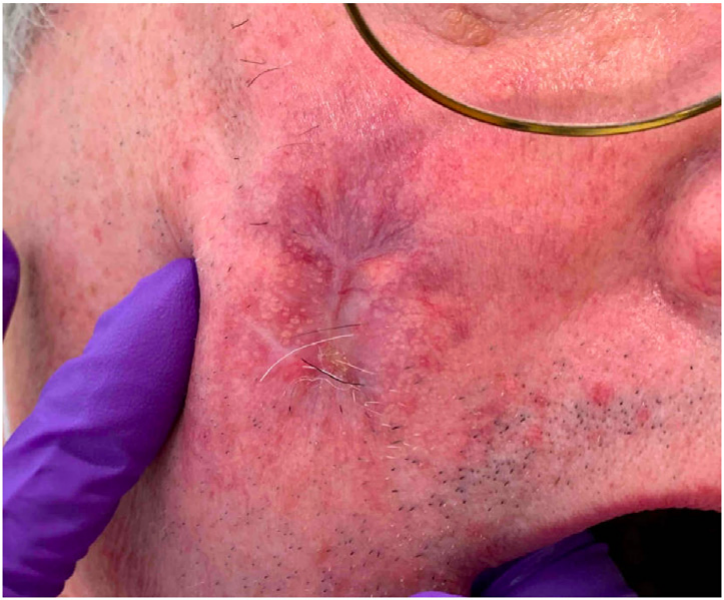
Complete healing of prior ulceration, now replaced with an atrophic scar with telangiectasias following combination topical MEK inhibition and methotrexate.

The epidermal component of the ulcerative nodules completely healed (Fig 4) and the nodular masses decreased in size over 15 months.

With topical trametinib administration, the patient developed an acneiform eruption on the lower face and anterior neck, without any other side effects of MEK inhibition. The patient has continued the combination of oral methotrexate, trametinib cream, and serial ILTAC without additional adverse events, and the subcutaneous tissue continues to soften with clinical near complete response.

## Discussion

We report a novel case of CRDD that recurred following surgical excision and was refractory to numerous lines of therapy, including ILTAC monotherapy, but ultimately responded to the addition of topical MEK inhibition and methotrexate.

While methotrexate alone or in combination with other agents may be effective for RDD,^7^ it has been less successful in patients with CRDD with only 2 complete responses^8^^,^^9^ noted out of 10 cases. While the methotrexate was likely the central driver of the patient’s deep nodular response given the thickness of the patient’s ulcerated plaques, more rapid superficial healing was noted with the topical MEK inhibitor prior to methotrexate initiation. The use of topical or systemic MEK inhibitors in combination with methotrexate has not been reported. The intralesional steroid alone did not halt progression. Thus, it appears the combination of methotrexate with topical trametinib contributed to healing.

In conclusion, CRDD can be progressive, multifocal, and particularly challenging to treat, as seen in our patient. Isolated CRDD may warrant early systemic therapy to prevent ulceration and disfigurement, especially when lesions present in cosmetically sensitive areas. Our case adds further support for the use of methotrexate in CRDD. While cobimetinib, an oral MEK inhibitor, has been successfully used in RDD and was approved by the Food and Drug Administration (FDA) in November 2022 for histiocytic neoplasms, further research of topical MEK inhibition in superficial CRDD is warranted. We recommend confirming mutation status when possible. Based on this patient’s case, combination methotrexate and topical MEK inhibition may be considered for treatment-refractory CRDD.

## Conflicts of interest

Nicole R. LeBoeuf is a consultant and has received honoraria from Bayer, Seattle Genetics, Sanofi, Silverback, Fortress Biotech, and Synox Therapeutics outside the scope of the submitted work.
